# Shapeshifting tau: from intrinsically disordered to paired-helical filaments

**DOI:** 10.1042/EBC20220150

**Published:** 2022-12-16

**Authors:** Kurtis Mengham, Youssra Al-Hilaly, Sebastian Oakley, Kamillia Kasbi, Mahmoud B. Maina, Louise C. Serpell

**Affiliations:** Sussex Neuroscience, School of Life Sciences, University of Sussex, Falmer, BN1 9QG, U.K.

**Keywords:** Alzheimer's disease, amyloid, intrinsically disordered proteins, liquid liquid phase separation, protein misfolding, tau

## Abstract

Tau is an intrinsically disordered protein that has the ability to self-assemble to form paired helical and straight filaments in Alzheimer’s disease, as well as the ability to form additional distinct tau filaments in other tauopathies. In the presence of microtubules, tau forms an elongated form associated with tubulin dimers via a series of imperfect repeats known as the microtubule binding repeats. Tau has recently been identified to have the ability to phase separate *in vitro* and in cells. The ability of tau to adopt a wide variety of conformations appears fundamental both to its biological function and also its association with neurodegenerative diseases. The recently highlighted involvement of low-complexity domains in liquid–liquid phase separation provides a critical link between the soluble function and the insoluble dysfunctional properties of tau.

## Introduction

**Figure 1:**
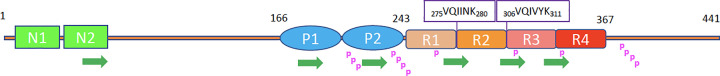
Schematic showing the major regions in the tau primary sequence This shows the two possible N terminal domains (N1, N2), two proline rich regions (P1, P2) and the imperfect repeat regions or microtubule binding regions: R1 (243-273), R2 (274-304), R3 (305-335) and R4 (336-367). Tau can exist with three or four repeats (3R or 4R, respectively) and R2 is missing from 3R tau. R2 and R3 contain hexapeptide motifs that have been highlighted in driving amyloidogenic propensity (VQIINK and VQIVYK). There are multiple possible phosphorylation sites on serine, threonine and tyrosine in tau and some of these are highlighted (purple p). NMR studies have shown that regions of the protein have β-sheet propensity (denoted by green arrows). Full-length tau 2N4R is shown.

Tau is a microtubule binding protein and an important component of the neuronal cytoskeleton as well as playing a role in the nucleus [1,2]. Humans have six major spliced isoforms in the central nervous system from MAPT, a single gene located on chromosome 17 (cytogenetic location 17q21.1), which range from 352 to 441 residues [3]. The longest form contains an N-terminal projection domain (residues 1-165), a proline rich region (residues 166-242), a microtubule binding region (MTBR) (residues 243-367) and a C-terminal (residues 368-441) and is referred to as 2N4R tau (Figure 1). The MTBR consists of four imperfectly repeated sequences: R1 (243-273), R2 (274-304), R3 (305-335) and R4 (336-367) [4] (Figure 1). Isoforms can differ in the number of microtubule-binding imperfect repeats and can exist in three or four repeat forms (3R and 4R, respectively) with there being equal amounts in the human cortex [5] (Figure 1). The primary sequence of tau has a relatively low proportion of hydrophobic amino acids (mean hydrophobicity of 0.4) [4,6] and has a high-number of polar and charged amino acid residues (net charge at neutral pH is +2), which results in it being highly soluble in water and natively unfolded. A large number of charged residues are found in the MTBR resulting in a local net-charge of +9 [4]. The primary structure of tau can contain an array of post-translational modifications of specific residues including: phosphorylation, acetylation, deamidation, methylation, O-glycylation or ubiquitination, many of which may impact on the structural organisation of the protein [6,7]. Truncation can also cause the loss of the N-terminal and/or C-terminal regions [8,9].

Intracellular accumulations of tau in tauopathies [10,11] arise when tau misfolds and self-assembles into filaments with the classic amyloid-like, cross-β structure [12]. The tauopathies include Alzheimer’s disease (AD), Pick’s-type frontotemporal dementia, cortical basal degeneration, chronic traumatic encephalopathy and progressive supranuclear palsy [13]. In AD, the filaments contain both 3R and 4R tau, while in Pick’s disease the filaments are composed of 3R only and in cortical basal degeneration, only 4R is deposited [13]. Despite the well-known association of tau misfolding with a large number of neurodegenerative diseases, it remains unclear how tau is involved in disease causation and progression and what may trigger protein misfolding. One potential area of interest is liquid–liquid phase separation (LLPS) where tau may be present at higher concentrations and this could give rise to the ideal conditions for abnormal protein aggregation [14]. In this mini-review, we discuss the shape-shifting nature of tau where it can interconvert from soluble and natively unfolded, to phase separated and cross-β assemblies. Here, we have mainly focussed on studies that did not include heparin to initiate assembly.

## The biological function and conformation of tau

**Figure 2:**
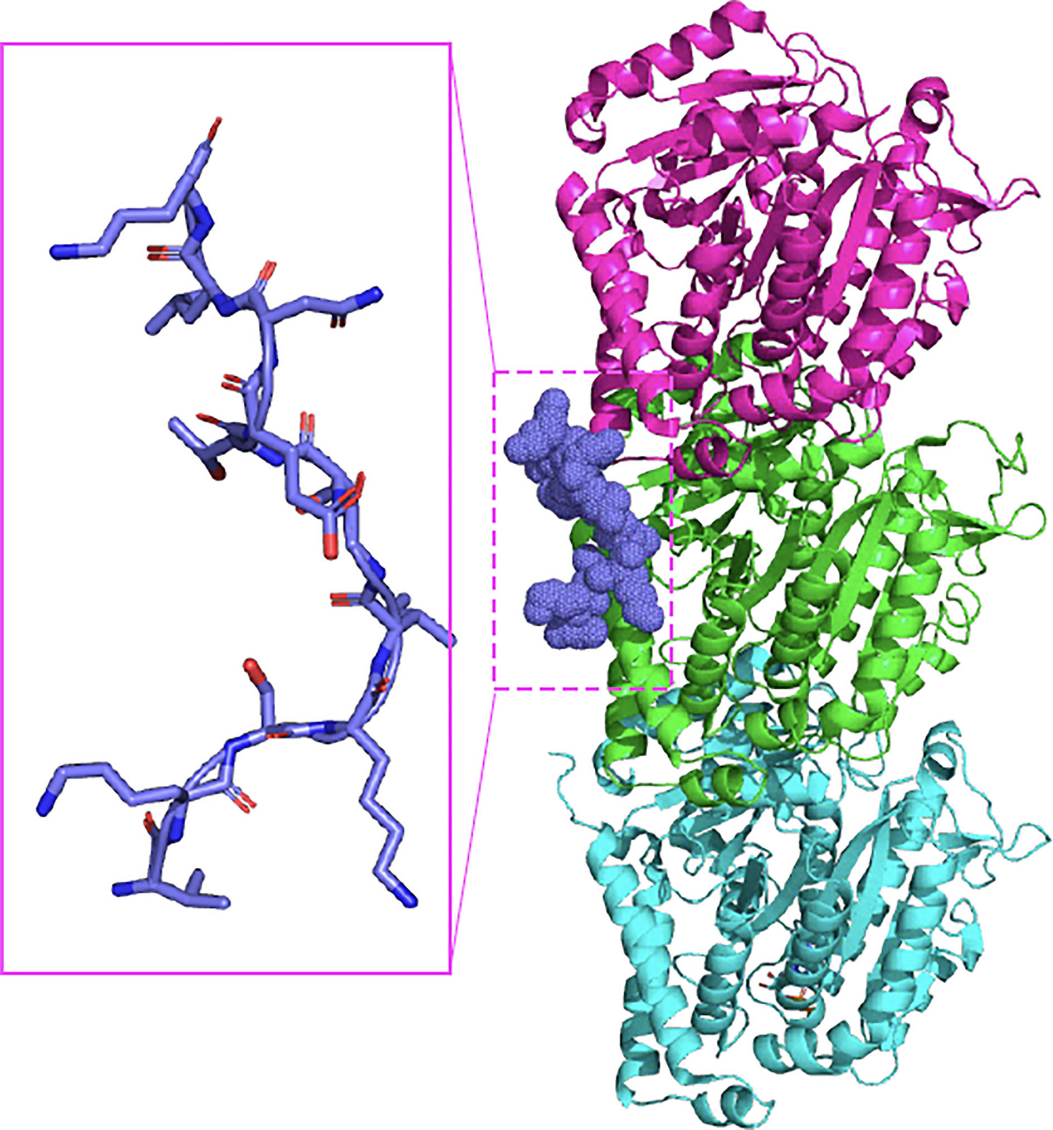
The structure of tau bound to microtubules Structure of microtubules showing the binding of tau between molecules solved using cryoEM [16] (6CVK .pdb). Insert shows the extended conformation of tau when it is associated with microtubules.

Tau is best known for its association and stabilisation of microtubules, predominantly in neuronal cells [15]. It binds to the tubulin dimers via the MTBR. The cryo-electron microscopy (cryoEM) structure of tau associated with microtubules revealed the high-resolution structure of R1-R2 regions organised like a staple across the tubulin molecules to stabilise the growing microtubules (6CVK .pdb) while R3-R4 and the proline-rich region were less well defined in the cryoEM map [16] (Figure 2). Modelling and experimental analysis of the full-length tau in association with microtubules revealed that post-translational modifications including phosphorylation and acetylation modulate the ensemble conformation resulting in altered binding affinities (7QPC) [17].

Although tau is named for its microtubule binding properties, it has been shown to bind to multiple proteins including actin and protein phosphatase 2A [18], various membrane proteins and α-synuclein [19]. More recently, tau has been identified in the nucleus [20] and further localised to the nucleolus where it appears to participate in chromatin remodelling [21]. Interestingly, the post-translational modifications on tau appear to regulate both its distribution, binding partners and also its conformation. Phosphorylation plays an important role in conformation of tau and nuclear tau is predominantly non-phosphorylated [1. 21]

Tau has no well-defined secondary or tertiary structure and is classified as an intrinsically disordered protein (IDP). Solution state NMR of full-length tau has been hampered by the large size and intrinsically disordered nature of the protein [22]. Circular dichroism studies conducted at different pH, temperatures and ionic strengths showed very little differences in the majority random coil secondary structure for 2N4R tau [4]. NMR studies [22] indicate that short regions of the tau sequence possess β-structure propensity, identifying previously known regions 274-284 and 305-315 which include the amyloidogenic VQIINK and VQIVYK motifs [23] with much of the rest of the protein being random coil in character (343-411) [23]. Serine, threonine or tyrosine phosphorylation have been suggested to modulate the amyloidogenicity of tau and most of the phosphorylation sites are found in the N-terminal portion of the protein mainly within the proline rich domains [22]. However, some potential phosphorylation sites are found in the repeat regions and phosphorylation of Y310 might be expected to affect self-assembly propensity since tyrosine 310 is found within the key amyloidogenic driving region.

## Self-assembly of tau

The mechanism of self-assembly of full-length tau into form amyloid fibrils remains unclear given the difficulty of enabling assembly *in vitro* without the contribution of additives. *In vitro*, full-length tau is robustly soluble, requiring additives to initiate self-assembly in contrast with the amyloidogenic nature of amyloid-β [24]. Additives that have been used include heparin [25], RNA [26] and lipids [27]. However, studies utilising the kinetics analysis in the absence of heparin pioneered by Linse et al. [28] have indicated that under quiescent conditions at pH8, tau 304-380(C322S) fragment assembles via a slow primary nucleation phase followed by a dominant secondary nucleation phase fuelled by the additional of monomers to the filament surfaces and the production of further oligomers arises from secondary nucleation events [28]. In general, self-assembly of tau peptides follows the expected secondary structural transition from random coil to β-sheet by circular dichroism measurements with time [29] although clear observation of the β-sheet signal contribution requires resuspension of a pellet due to the dominant nature of the random coil signal in solution [29]. This appears to indicate that assembly is slow and that there is a dynamic equilibrium that exists between fibrillar and soluble tau.

Historically, regions covering R1-R4 have been investigated, referred to as K18 (244-372) and K19 (244-372 lacking R2 275-305) using heparin to template aggregation [30,31], and short hexapeptides with sequences _306_VQIVYK_311_ [32] and _275_VQIINK_280_ [33] have been shown to form highly ordered fibrils *in vitro*. However, the inclusion of heparin appears not to result in filaments that resemble those extracted from AD brain [34,35] and isolated short peptides form steric zippers and do not provide information on intermolecular interactions of the larger fragments [32,36] (2ON9 .pdb & 5V5C .pdb, respectively).

**Figure 3:**
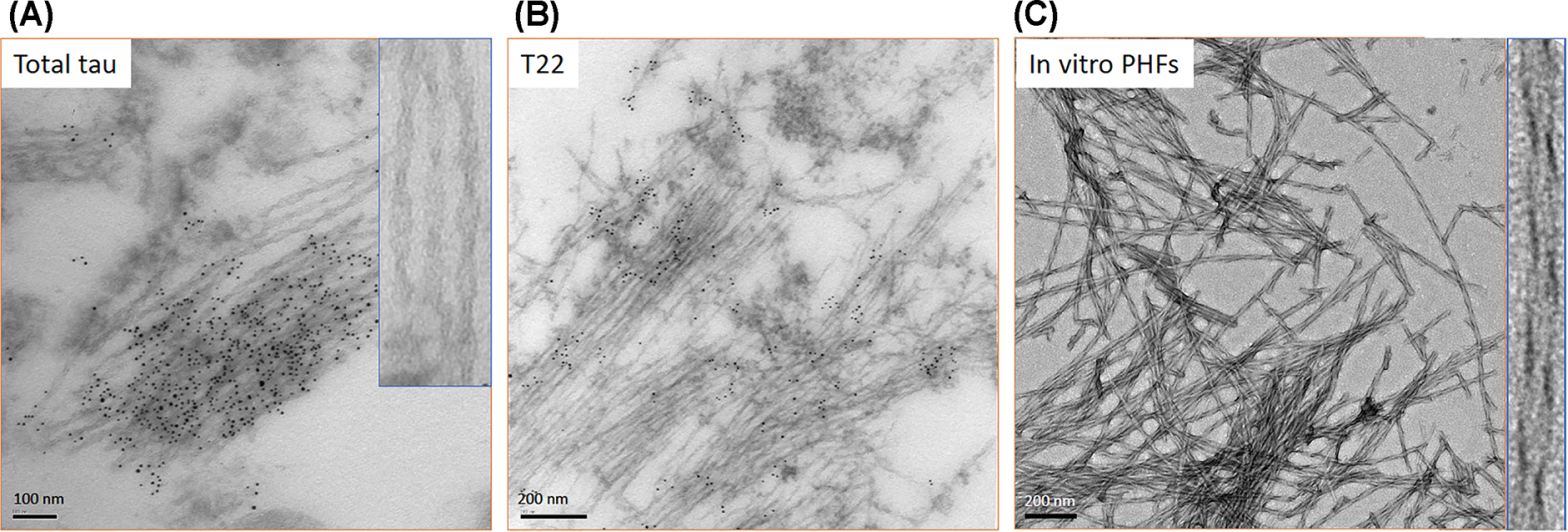
The morphology of paired helical filaments formed by tau *in vivo* and *in vitro*. Left and middle panel show immunogold labelling electron microscopy of tau filaments in AD brain tissue (tau filaments identified using antibodies against total tau (**A**) and tau oligomers (T22) (**B**) while the right panel (**C**) shows PHF formed in vitro from the tau fragment 297-391, dGAE [ 29]. Inserts show enlarged images to highlight the paired-helical appearance. Scale bars are shown.

Early work isolated a fragment of tau from AD brain which was resistant to degradation and this was mapped to 297-391 and named dGAE for the last three amino acids of the C-terminus of the fragment [8,9]. dGAE is able to self-assemble *in vitro* without any additives [29]. Under certain conditions, dGAE can form filaments that appear to twist and resemble PHFs by electron and atomic force microscopy and they share the amyloid cross-β structure by X-ray fibre diffraction [29,37] (Figure 3). Monitoring assembly using circular dichroism shows a gradual loss of random coil content and a concurrent increase in β-sheet signal [29]. dGAE contains a single cysteine residue at 322 and oxidation to form disulphide linked dimers hinders assembly [29]. In contrast, previous studies using different tau models, such as full-length tau and K18/K19 fragments, indicated that these disulphide linked dimers are essential for tau self-assembly [38–40]. These contrasting findings illustrate the importance of truncation and the influence of the environment on tau self-assembly, and highlights the need for a reliable model in tau self-assembly. The region first observed by cryoEM of Alzheimer’s derived tau filaments, region 306-378 [41], is also able to self-assemble *in vitro* and to seed the assembly of full-length tau [42]. Following oxidation of dGAE, one of the resulting modifications is the formation of dityrosine cross-links. These cross-links appear to ‘freeze’ or halt further self-assembly of dGAE and prevent further elongation [43,44]. Dityrosine cross-linking is observed in neurofibrillary tangles where it may stabilise the deposits protecting them from disassembly, while only soluble forms of tau appear to be susceptible to cross-linking [44].

Early fluorescence resonance energy transfer (FRET) studies of tau in solution have suggested interactions between the N and C-termini of tau suggesting the formation of a ‘hairpin’ [45]. More recent studies have identified two stable forms of tau from brain and recombinant sources, one of which is inert and the other is assembly competent [46]. Although no obvious differences in secondary structure were apparent, the two forms differed in intermolecular contacts involving lysine residues located in R1 and R2 and modelling studies supported by protease digestion experiments implicated the exposure of the assembly driving regions VQIINK and VQIVYK in repeat 2 and repeat 3, respectively (Figure 1) in the assembly competent form, while it was buried in the assembly inert form [46]. Interesting, the inert form could be converted to assembly competent form in the presence of heparin. This highlights the capacity of tau to adopt alternative structural ensembles capable of multiple functions, from molecular interactions to self-assembly.

## The pathological structures of tau

**Figure 4:**
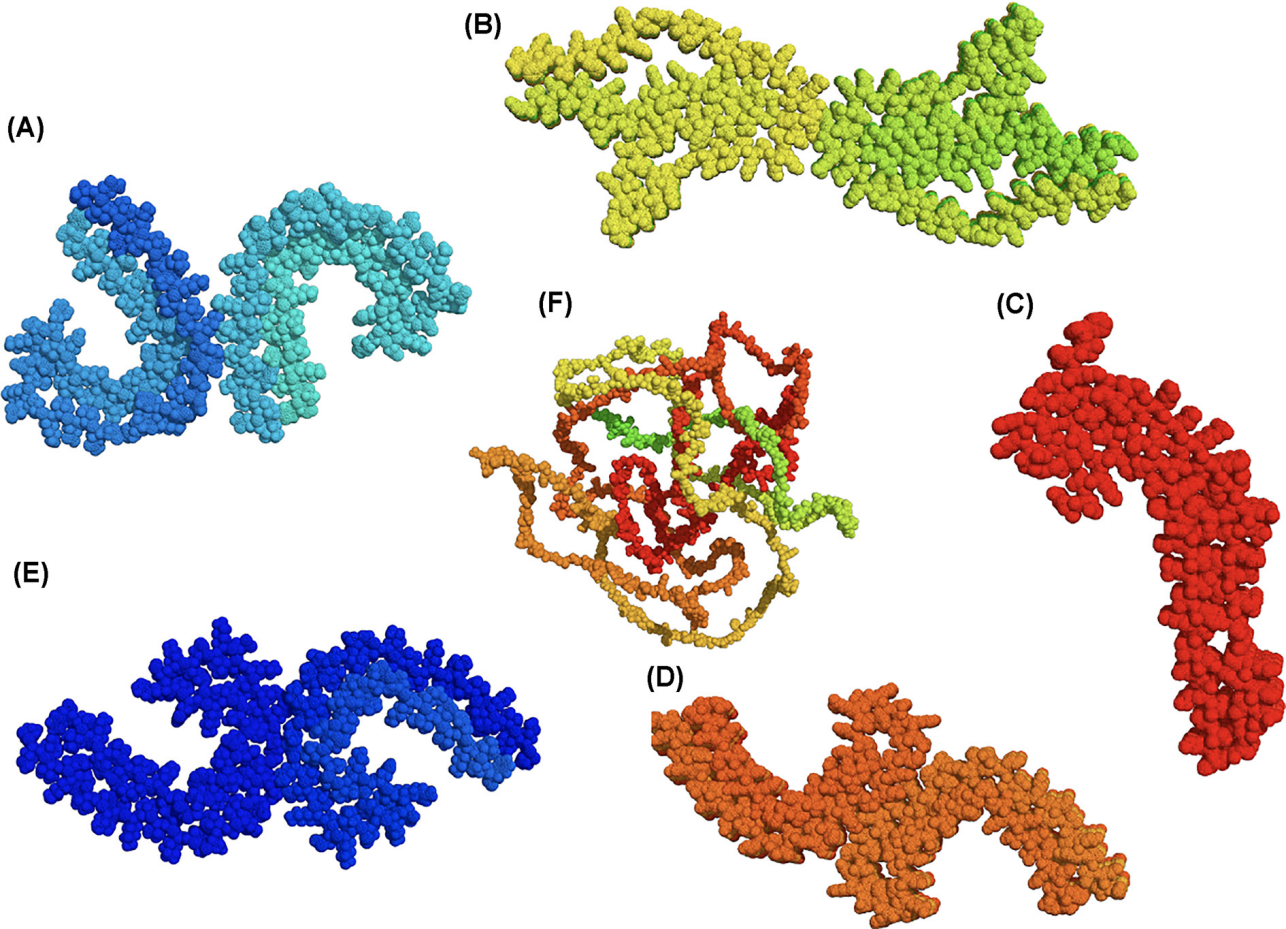
Structures of tau CryoEM structures for tauopathy derived filaments showing the conformation of the building blocks viewed down the fibre axis using spheres for the atoms. (**A**) Straight filaments (SF) from AD brain [48] (6HRF), filaments from cortical basal degeneration [49] (6TJX), (**C**) filaments from Pick’s disease [50] (6GX5), (**D**) filaments from chronic traumatic encephalopathy [51] (6NWQ), (**E**) paired helical filaments from AD brain ([48] (6HRE) and (**F**) shows a predicted structure of 2N4R using alphafold (https://alphafold.ebi.ac.uk) [52].

Electron microscopy of tissue sections clearly shows the paired helical appearance of AD filaments with a regular repeat distance of 73 nm (Figure 3). AD tissue extracted filaments provided more detail with a C-shaped cross-section and the identification of a fuzzy coat which could be removed using proteases [47]. Early work using X-ray fibre diffraction revealed that paired helical filaments (PHF) isolated from AD brains shared the cross-β structure that characterises amyloid fibrils [12] demonstrating that the tau PHF share the hallmark cross-β amyloid structure and leading to the classification of tau filaments as amyloid fibrils. More recently, major advances in cryoEM techniques have resulted in a gallery of exciting details of the different architectures of disease associated tau filaments. All the structures are cross-β, with a distance of 4.7 Å between stacked β-strands but the organisation of each individual monomeric cross-section differs. Starting with AD PHF and straight filaments (SF), it was shown that the core structure was made up of repeats 3R-4R, residues 306-378 [41] later extended to 304-380 [48]. It was confirmed that the filament cross-section is made up of two C-shaped protofilaments that associate back-to-back and have slightly different protein–protein interactions for PHF versus SF (Figure 4). Chronic traumatic encephalopathy extracted filaments share a similar structure to those of AD, while Pick’s disease filaments are quite different and arise from only 3R tau (Figure 4). On the other hand, cortical basal degeneration filaments are made of only 4R tau and give another architecture again (Figure 4). Since these early structures were solved, many more tauopathy filaments have been provided all with similarities and differences raising the pressing question of what drives and then determines the specific amyloid fold and how much the conformation and architecture impact on the characteristic symptoms of each disease. Solid-state NMR of filaments formed from full-length tau 2N4R in the absence of heparin revealed that the region P270-S400 was highly structured in the core and not observed by ^1^H-^15^N INEPT, which allows observation of dynamic regions [49], suggesting a larger core region formed by this isoform *in vitro* that those derived from AD tissue and more similar to those observed for filaments extracted from cortical basal degeneration tissue [22,49,50].

**Figure 5:**
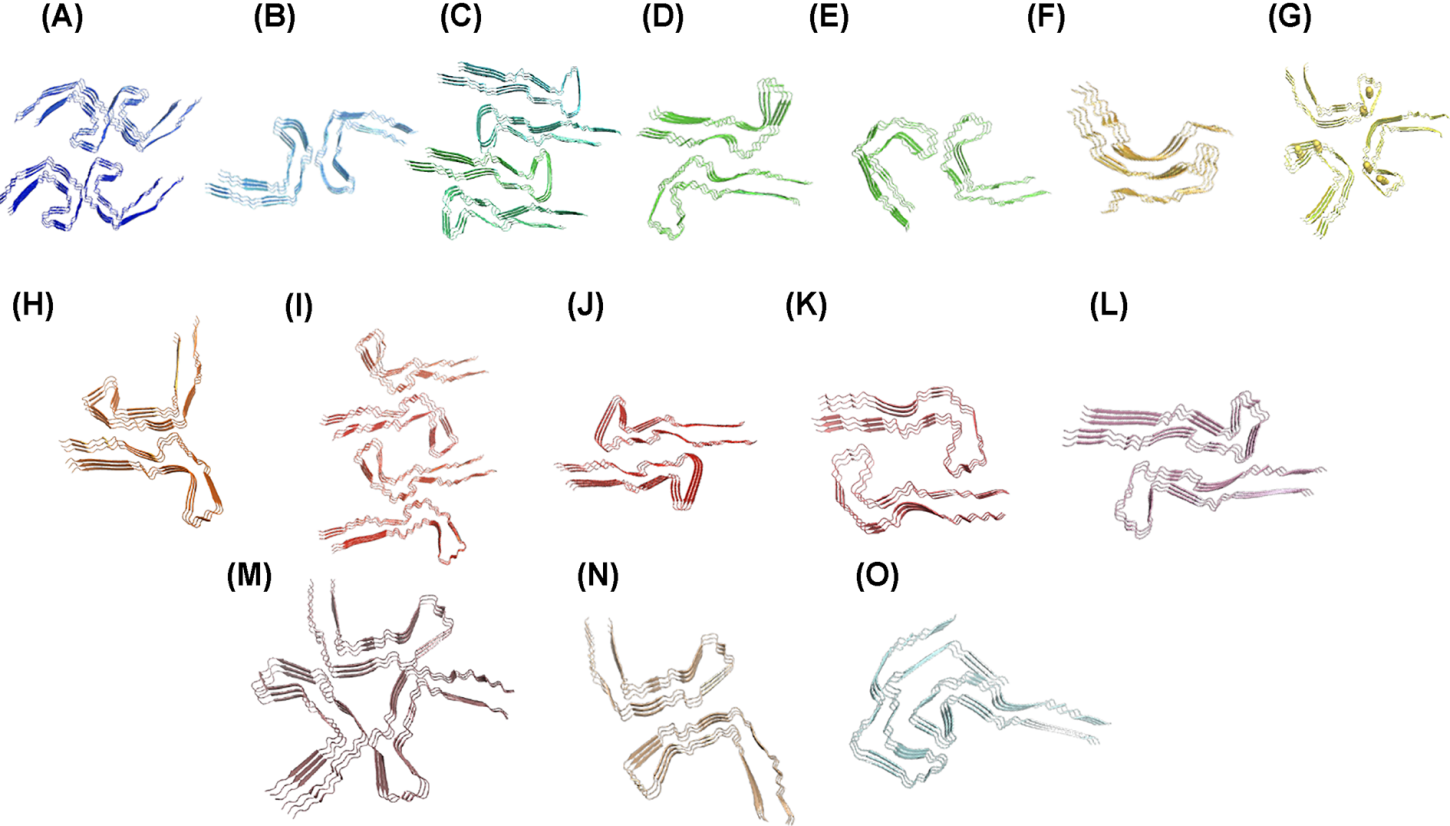
Comparison of the structures of tau fragments formed *in vitro* CryoEM structures for tau297-391, dGAE and 266/297-391 [54] assembled under a wide variety of conditions. Listed conditions refer to those entered in table 1 of Lövestam et al. [54] and identified by conditions in brackets. PDB files are provided for each structure and all conditions are 10 mM PB, 10 mM DTT, pH 7.4, 200 rpm and differences are noted in square brackets. (**A**) 7QJV (2c) 297-391 10 mM PB, 10 mM DTT, pH 7.4, 200 rpm; (**B**) 7QJW (8a) 266/297-391 [200 mM NaCl]; (**C**) 7R5H (10b) 266/297-391 [200 mM KCl]; (**D**) 7QJY (9a) 266/297-391 [200 mM LiCl]; (**E**)7QJZ (9b) 266/297-391 [200 mM LiCl]; (**F**) 7QKF (12a) 266/297-391 [200 μM CuCl2]; (**G**) 7QK5 (10a) 266/297-391 [200 mM KCl, 200 rpm; (**H**)7QKU (14a) 266/297-391 [20 mM MgCl_2_ 100 mM NaCl]; (**I**) 7QKJ (14b) 266/297-391 [20 mM MgCl_2_ 100 mM NaCl]; (**J**) 7QKL (11a) 266/297-391 [100 μM ZnCl_2_]; (**K**) 7QL3 (8b) 266/297-391 [200 mM NaCl]; (**L**) 7R4T (16a) 266/297-391 [10 mM NaHCO_3_ 100 mM NaCl]; (**M**) 7QKV (15a) 266/297-391 [10 mM MgSO_4_ 100 mM NaCl]; (**N**) 7QKX (15b) 266/297-391 [10 mM MgSO_4_ 100 mM NaCl]; (**O**) 7R5H (10b) 266/297-391 [200 mM KCl].

Recent advances have been made in analysis of atomic force microscopy images allowing single filaments to be examined and reconstructed paving the way for the potential to interrogate the structure of individual filaments within a sample [53]. This approach was used to investigate the structure of PHFs generated without additives from dGAE *in vitro* (Figure 3), indicating that these filaments shared a structure with those extracted from AD brain [37,48,53]. More recently, Lovestam et al. confirmed that the dGAE has the ability to form AD-like PHF, solving the structure by cryoEM [54] and revealing multiple different filament conformations (Figure 5). Interestingly, small changes in the assembly conditions can modulate the conformation and architecture of the filaments including an influence of shaking speed and addition of various salts (Figure 5). These results point to the important contribution of environmental conditions in generating disease specific tau filament structures and highlighting the range of structural strains possible [54]. Extension of the peptide at the N- and C-termini resulted in inhibition of assembly suggesting that these regions are important in maintaining the solubility of full-length tau. The effect of pseudophosphorylation (Ser/Thr>Asp) was also explored and it was observed that pseudophosphorylation at C-terminal sites (396-404) overcame the inhibitory effect of the C-terminal region resulting in formation of PHFs [54].

## Liquid–liquid phase separation: connecting the dots?

The mechanism by which tau transitions from a highly soluble, flexible protein to an ordered amyloid fibril remains unclear. Tau sequence is defined by the primary sequence which contains low-complexity domains which are often enriched in polar and charged amino acids (Gln, Ser, Pro, Glu and Lys) [55]. They also contain a low number of hydrophobic amino acids (Val, Leu, Ile, Met, Phe, Trp and Tyr) which would normally form the core of a globular protein via hydrophobic collapse [55]. Tau shares some sequence characteristics with prion-like domains and proteins implicated in LLPS, such as the RNA binding proteins FUS, TDP43 and hnRNPA1. LLPS is an emerging phenomenon which results in dynamic and reversible structures that are known as membraneless organelles, condensates, liquid droplets and coacervates since the phase separation is driven by interactions within the droplet that result in a different phase from the surrounding solvent environment. Tau has been observed to associate with phase separated organelles in the cytoplasm and in the nucleus [56] and recently LLPS has been implicated in the mechanism of nucleation of microtubules [57]. Tau has also been observed in stress-granules in association with T-cell intracellular antigen (TIA1), which maintain partially translated mRNA under conditions of stress [58] and in other membraneless organelles [59,60]. The ability of tau to associate with nucleic acids may arise from its function observed in the nucleolus [21]. TIA1 is important in generation of stress granules and tau has been reported to bind TIA1 [61,62], while reduction of TIA1 has been shown to reduce tau-mediated neurodegeneration *in vivo* suggesting a key important interaction occurs on the pathological pathway in stress granules [63]. Furthermore, TIA1 has been implicated in the formation and propagation of toxic oligomeric tau [64].

**Figure 6:**
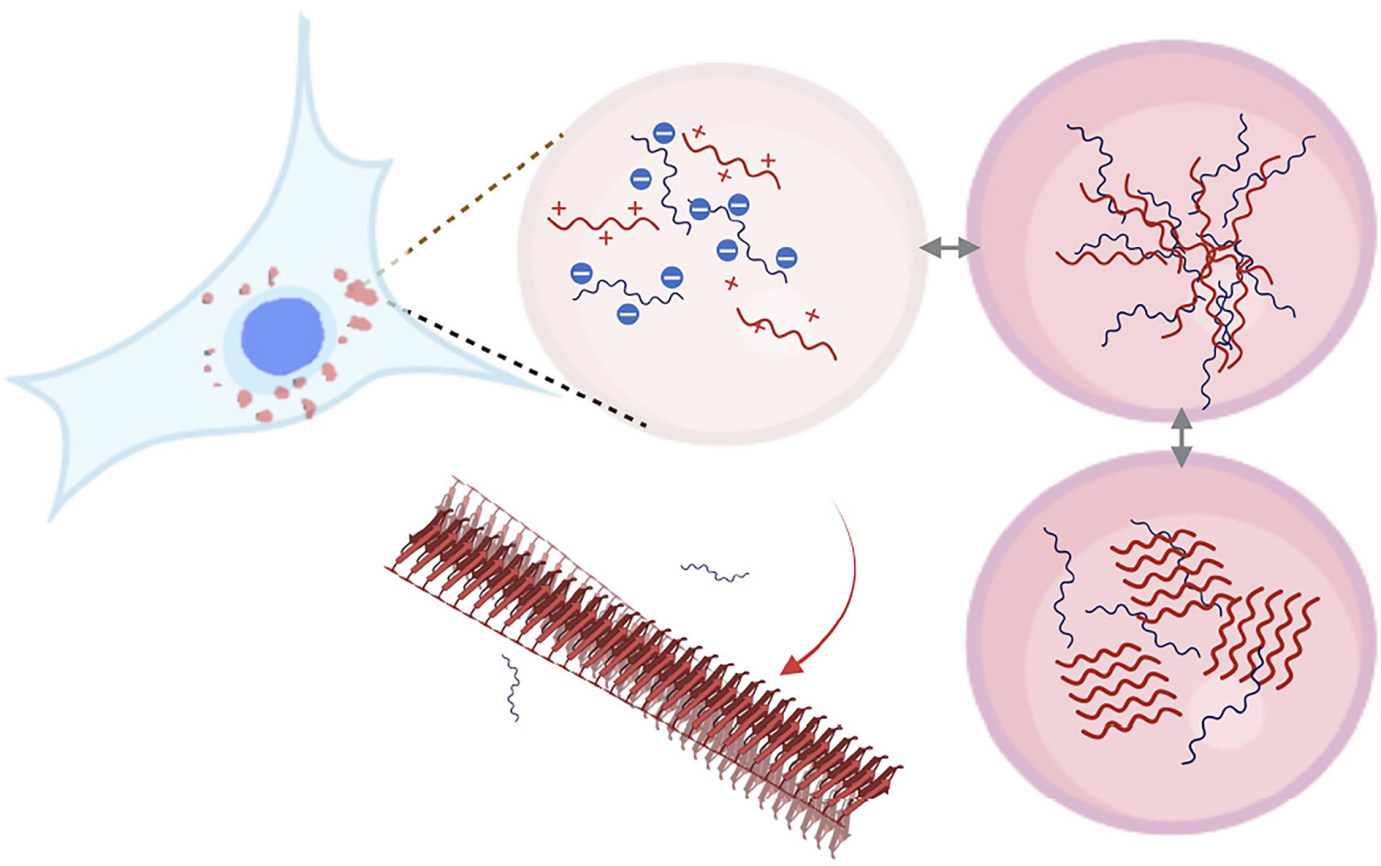
A potential pathway to tau amyloid Schematic image showing the potential for transition from liquid–liquid phase separation to solid phase amyloid fibrils in liquid droplets in cells and showing coacervation driven by charge-charge interactions between tau (red) and RNA (blue).

*In vitro* studies of tau have revealed the ability to form LLPS in association with crowding agents or RNA [65,66] (Figure 6). The conformation of tau within the condensates remains unclear since these membraneless organelles are thought to behave as hydrogels. Under normal conditions, phase separation is highly dynamic and fully reversible. However, a transition to the (largely irreversible) insoluble amyloid state has been observed for other neurodegenerative disease related proteins such as FUS in amyotrophic lateral sclerosis [67] and increasingly discussed in relation to other neurodegenerative disease proteins [68,69]. Tau has been shown to associate with RNA, a cofactor known to increase aggregation [26], and phase separate into a complex coacervate that is dynamic allowing monomeric tau to move freely [65] and reversibly [66]. In cells, GFP labelled tau441 formed intraneuronal droplets which show fluorescence recovery after photobleaching (FRAP) implying a liquid state of the structures [73]. The GFP-tau441 was expressed in a phosphorylated form or incubated in crowding agents and was then shown to form liquid droplets in cells [73]. *In vitro* phosphorylated tau droplets matured with time and formed fibrillar structures providing some support for the idea that LLPS may be an initiator of transition to the insoluble amyloid state [70]. Phase separation of tau can be optimised by titration of RNA and salt (NaCl) concentrations and examination of the regions of tau that participate in RNA-binding centred within proline rich and MTB domains [62]. Furthermore, the inclusion of TIA1 resulted in LLPS of tau without addition of crowding agents and this appeared to support further self-assembly to form oligomeric and fibrillar tau [62].

The conditions that result in conversion of tau from LLPS to filaments remains to be further explored in detail. Structural details of tau in LLPS and during transition are still lacking. However, evidence is accumulating for LLPS providing the ideal environment for the conversion of other neurodegenerative disease proteins from soluble to amyloid-structured and this may provide the missing piece of the puzzle for tau aggregation *in vivo*. Whether LLPS is a required intermediate or just another potential pathway is yet to be shown but it may unlock future therapeutics and advances in the treatment of tauopathies and other neurodegenerative diseases.

## Summary

- Tau is a protein that is central to a large number of neurodegenerative diseases collectively known as tauopathies and understanding the conformational space occupied by tau is essential for deciphering the mechanisms of pathological tau in tauopathies.
- Membraneless organelles arising from liquid liquid phase separation in cells may provide a suitable environment for initiation of aggregation prone conformations of tau resulting in misfolding, aggregation and deposition.

## Open Access

Open access for this article was enabled by the participation of University of Sussex in an all-inclusive *Read & Publish* agreement with Portland Press and the Biochemical Society under a transformative agreement with JISC.

## Abbreviations

AD: Alzheimer's disease
cryoEM: cryo-electron microscopy
FRAP: fluorescence recovery after photobleaching
FRET: fluorescence resonance energy transfer
LLPS: liquid–liquid phase separation
MAPT: Microtubule associated protein tau
MTBR: microtubule binding region
PHF: paired helical filaments
SF: straight filaments
TIA1: T-cell intracellular antigen
